# Medical College Association

**Published:** 1878-07

**Authors:** 


The Medical College Association.—The Association of American Medical Colleges met in the hall of the Buffalo Medical College, June 3, 1878. The President, Prof. J. B. Biddle, of the Jefferson Medical College, occupied the chair, and Dr. L. Connor, of the Detroit Medical College, was at his desk as Secretary.

The first order of business being the presentation of credentials, the following colleges were found to be represented:

Rush Medical College—Prof. Moses Gunn.

Jefferson Medical College—Profs. S. D. Gross and J. B. Biddle.

Medical Department University of Louisville—Prof. J. M. Bodine.

Detroit Medical College—Profs. L. Connor and E. W. Jencks.

Chicago Medical College—Prof. N. S. Davis.

Miami Medical College—Prof. W. H. Mussey.

Starling Medical College—Prof. H. G. Landis.

Medical Department of University of Nashville and Vanderbilt—Profs. T. Menes and W. T. Briggs.

Louisville Medical College—Prof. A. B. Cook.

Medical Department University of Iowa—Profs. E. F. Clapp, W. F. Peck, W. S. Robertson.

Kansas City College of Physicians and Surgeons—Prof. T. B. Lester.

Medical Deprrtment of Michigan University—Prof. E. S. Dunster and George E. Frothingham.

Missouri Medical College—A. P. Lantford.

Bellevue Hospital Medical College—Prof. A. Flint, Jr.

The minutes of the previous meeting having been published, the reading of them was dispensed with.

Objections against the admission of Howard University, D. C., to the Association, were then taken up, and after some discussion the objections were sustained, and the college refused admission by a vote of 12 to 2.

The Secretary presented a report, from which it is learned that there are twenty-five regular members and one affiliated member. Applications for membership had been received from the Ohio Medical College, March 26th, and Alabama Medical College, March 18th last. As soon as the report was issued last fall, a letter was sent to all regular medical colleges of the United States, asking if they conformed to the articles of confederation required of regular or affiliated members. Accompanying this letter was sent the pamphlet containing a history of the organization of the Association, its constitution, by-laws, articles of confederation, and list of members. Two colleges—Harvard, and the Medical Department of the University of Pennsylvania—replied that they regarded it unadvisable for them to join the Association.

Annual reports from the colleges were then received and read by the Secretary. These reports noted the honorary degrees conferred by each college within the year, with the age and name of the recipient, and the reason why the degree was given. They also gave the names of all persons who had been allowed remissions or reductions of established fees, with the reasons in each case for the proceeding.

The Secretary also presented from the same colleges catalogues and advertisements issued by the same call as during 1877–78.

The following resolution was then offered by Prof. Flint, of New York:

*Resolved*, That the Secretary of the Association be, and is hereby directed, to furnish, one in each year, to each and every college member and to each affiliated college, a printed list of college members and affiliated colleges, the diplomas and tickets of which are to be recognized by the college members and affiliated colleges; and also to furnish to college members and affiliated colleges, a printed list of those colleges (not including irregular colleges) of the United States that are not affiliated, and that are not eligible for membership of the Association, the diplomas and tickets of which are not to be recognized by college members and affiliated colleges; and also to furnish with said list of colleges not to be recognized, the dates at which said colleges had become ineligible for membership of the Association, and after which the diplomas and tickets of said college are not to be recognized.

This was adopted with slight modification.

A resolution was offered by Prof. Flint, seconded by the College of Physicians and Surgeons in New York, to change the number of beneficiaries from five per cent. of matriculants to ten per cent. of graduates.

It was laid over one year.

AFTERNOON SESSION.

On motion, Profs. Davis, Flint and Gross were appointed by the chair a committee to consider the whole matter in relation to the classification of the medical colleges.

Prof. Gross, with a few introductory remarks, offered a series of preambles and resolutions contemplating a meeting, in September, of representatives at Washington, for the purpose of raising the uniform standard of medical education.

Prof. Davis, in seconding the resolutions, gave a history of the efforts made to improve college-instruction during the past twenty-five years. He thought that sentiment in and out of the profession had reached a point which called for the proposed advancement in medical education. He was in favor of having a three-years' course of instruction, of not less than eight months' duration per year, and no student could enter upon his duties in any college until he had given some evidence of preparation. He hoped to live to see the medical profession at the head of all science, where it belonged, and the adoption of the resolutions would be an important step in the right direction.

Prof. Gunn, of Rush College, was in favor of a three-years' course as the requisite to graduation of students.

The Secretary read a letter from Prof. Seely, of the Ohio Medical College, in which he spoke in favor of a full course. The Secretary also stated that he was of the opinion from the correspondence he had had, that a majority of the colleges were in favor of the full course.

Prof. Bodine offered an amendment in effect that the conference be under the auspices of the Association. After some discussion, the amendment was lost.

The Friday preceding the meeting of the American Medical Association next year was fixed as the day for the holding of the conference, and the preamble and resolutions were then adopted.

Prof. Flint offered a preamble and resolution in effect that the tickets and diplomas of the Nashville Medical College shall not be recognized by the Association so long as the institution gives two graduating courses a year, and accepts three years' practice in lieu of a course of lectures.

The election of officers resulted as follows:

*President*—J. B. Biddle, M.D.

*Vice-President*—N. S. Davis, M.D.

*Secretary and Treasurer*—Laertes Connor, M.D.

Dartmouth Medical College offered its resignation as an active member of the Association. The matter was laid on the table for one year.

The medical college at Ft. Wayne having been dissolved, and a new one organized, the application of the latter to the place of the former was refused. The new organization was directed to send in its formal application as a new college.

The College of Physicians and Surgeons of Indianapolis, and the Medical Department of the University of Missouri signified their intention of applying for membership in the Association.

On motion of Prof. Gross, the thanks of the Association were tendered to the officers for the efficient manner in which they had discharged their duties.

On motion of Prof. Connor, the Association tendered its hearty thanks to the officers and faculty of the Buffalo Medical College for their courtesy in furnishing commodious rooms for the meeting of the Association.

The Association then adjourned, subject to the call of the President.—*Extract from Report of Buffalo Commercial Advertiser.*

